# Investigating the Control of Chlorophyll Degradation by Genomic Correlation Mining

**DOI:** 10.1371/journal.pone.0162327

**Published:** 2016-09-12

**Authors:** Frederick P. Ghandchi, Gustavo Caetano-Anolles, Steven J. Clough, Donald R. Ort

**Affiliations:** 1 Institute for Genomic Biology, University of Illinois at Urbana-Champaign, Urbana, IL, United States of America; 2 Department of Crop Sciences, University of Illinois at Urbana-Champaign, Urbana, IL, United States of America; 3 Soybean/maize Germplasm, Pathology, and Genetics Research Unit, USDA/ARS, University of Illinois at Urbana-Champaign, Urbana, IL, United States of America; 4 Department of Plant Biology, University of Illinois at Urbana-Champaign, Urbana, IL, United States of America; 5 Global Change and Photosynthesis Research Unit, USDA/ARS, University of Illinois at Urbana-Champaign, Urbana, IL, United States of America; Stony Brook University, UNITED STATES

## Abstract

Chlorophyll degradation is an intricate process that is critical in a variety of plant tissues at different times during the plant life cycle. Many of the photoactive chlorophyll degradation intermediates are exceptionally cytotoxic necessitating that the pathway be carefully coordinated and regulated. The primary regulatory step in the chlorophyll degradation pathway involves the enzyme pheophorbide a oxygenase (PAO), which oxidizes the chlorophyll intermediate pheophorbide a, that is eventually converted to non-fluorescent chlorophyll catabolites. There is evidence that PAO is differentially regulated across different environmental and developmental conditions with both transcriptional and post-transcriptional components, but the involved regulatory elements are uncertain or unknown. We hypothesized that transcription factors modulate PAO expression across different environmental conditions, such as cold and drought, as well as during developmental transitions to leaf senescence and maturation of green seeds. To test these hypotheses, several sets of *Arabidopsis* genomic and bioinformatic experiments were investigated and re-analyzed using computational approaches. PAO expression was compared across varied environmental conditions in the three separate datasets using regression modeling and correlation mining to identify gene elements co-expressed with PAO. Their functions were investigated as candidate upstream transcription factors or other regulatory elements that may regulate PAO expression. PAO transcript expression was found to be significantly up-regulated in warm conditions, during leaf senescence, and in drought conditions, and in all three conditions significantly positively correlated with expression of transcription factor *Arabidopsis thaliana* activating factor 1 (ATAF1), suggesting that ATAF1 is triggered in the plant response to these processes or abiotic stresses and in result up-regulates PAO expression. The proposed regulatory network includes the freezing, senescence, and drought stresses modulating factor ATAF1 and various other transcription factors and pathways, which in turn act to regulate chlorophyll degradation by up-regulating PAO expression.

## Introduction

Chlorophyll is a central molecule in plants that is essential to photosynthesis in absorbing light and transferring excitation energy, a portion of which is ultimately captured in plant biomass. Chlorophyll synthesis and breakdown are two metabolically significant processes of higher plants that can have significant economic consequences for crop agriculture. While the intricate chlorophyll biosynthetic pathway is well understood, basic knowledge about the chlorophyll degradation machinery and its regulation is uncertain. The chlorophyll degradation pathway consists of several enzymes that convert chlorophyll to non-fluorescent chlorophyll catabolites (NCCs), leading to loss of green color and absorption of visible light [1,2].

The key controlling enzyme involved in the chlorophyll degradation pathway is pheophorbide *a* oxygenase (PAO), a Rieske-type iron-sulfur protein and monooxygenase that activates molecular oxygen with its mononuclear iron center, functioning as an important cofactor in the reaction [3]. PAO oxidizes the degradation pathway intermediate pheophorbide a to red chlorophyll catabolite (RCC) by opening and linearizing the porphyrin ring leading eventually to the production of NCCs. As a rate controlling step of the pathway, PAO is expected to be under tight regulation [4].

A role for transcriptional regulation of the chlorophyll degradation pathway is suggested by several findings [5]. For example, evidence for the involvement of coordinated transcriptional regulation of plant chlorophyll levels include the clustering and co-regulation of gene expression patterns of stay-green (SGR), chlorophyllase b, non-yellow coloring (NYC), PAO and other enzymes or related proteins involved in the chlorophyll degradation pathway [4,6,7]. Environmental factors also modulate the chlorophyll degradation pathway, such as exposure to freezing temperatures in seeds inhibiting chlorophyll breakdown, which was also shown to be connected to post-translational dephosphorylation and activation of PAO [8].

The chlorophyll degradation pathway is relevant to the agricultural sector by modulating postharvest fruit and vegetable color [9–11]. Disruption of the pathway results in the “green seed problem” of oilseed crops such as canola and soybean, leading to photoactive chlorophyll contaminated oils, which in turn cause rancidness and financial losses [12,13]. In addition, optimizing chlorophyll levels in crop canopies could improve canopy photosynthetic efficiency, leading to higher crop yields [14]. Finally, delaying the induction of pathway in leaves by installing a “stay-green” trait could improve crop productivity, by taking advantage of the lengthening growing season brought on by global warming [15–17]. This study identifies upstream regulatory elements of PAO, which can be used to modulate plant response to biotic and abiotic factors and thus address wider issues in agribusiness.

## Materials and Methods

### Microarray Datasets

Three experiments on the analysis of global gene expression under conditions that should influence PAO gene expression in *Arabidopsis thaliana* were chosen as the expression datasets to mine for genes that co-expressed with PAO. These transcriptomic experiments were conducted using the Affymetrix ATH1 microarray chip technology containing gene expression data for 22,810 predicted genes and downloaded from the NIH GEO database [18]. The GSE55907 dataset originated from freezing tolerance induction experiments (4°C for 24 hrs) or warm (22°C) conditions on wild-type Columbia (Col-0) and several transgenics that were over-expressing genes associated with cold responses: a C-repeat binding factor (CBF), heat shock transcription factor C1 (HSFC), and a DREB and EAR Motif protein (DEAR) [19]. The GSE5727 dataset originated from leaves sampled during and prior to senescence, and were performed on wild-type Col-0, a jasmonic acid (JA) coi1 mutant, an ethylene signaling pathway ein2 mutant, and a NahG transgenic that would dampen salicylic acid (SA) signaling pathways [20]. The GSE72050 dataset originated from a study on the effect of drought on global gene expression in leaf tissue from wild-type Col-0 and NAC26-transcription factor overexpressing lines [21]. Microarray data were formatted for statistical analysis using the Python scripting language such that all data were normalized and log2 transformed in a similar manner.

### Statistical Analysis

Statistical and correlation analysis were performed on each microarray dataset using statistical programming language scripts of the statistical analysis system (SAS), written and tailored specifically for this project (S1 Appendix). PAO expression levels were compared within each microarray dataset among differing plant lines and environmental conditions using analysis of variance (ANOVA). Following this, PAO expression was modeled by other genetic elements using multiple linear regression (MLR) performed by automation in either forward method to add gene variables at a P = 0.05 cutoff, or in a stepwise method to both add and remove gene variables at a P = 0.05 cutoff. This was done until a significant model predicted PAO levels and R^2^ = 1, meaning that the model explained the entire variation in the PAO expression. Based on functional analysis results, particular upstream regulatory candidates were analyzed for co-expression with PAO using correlation analysis and general linear model (GLM). Upstream regulatory candidates and PAO were compared side-by-side among plant lines and environmental conditions using frequency analysis.

### Functional Classification

Functional determination of upstream regulatory candidates was accomplished using two unique, complementary methods. The first method involved the relational database structured query language (SQL) native functionality within the SAS statistical programming language. Pre-stored gene ontology data were crossed with the shortened co-expressed gene list using a custom-made SQL query to determine the functions of these genes and whether they belonged to any transcriptional factor families of interest. The second method relied on the Database for Annotation, Visualization and Integrated Discovery (DAVID) analysis tool [22], which utilized a unique algorithm to pool together information about large inputted gene lists based on different functional annotation tools including gene ontology (GO) terms, the Kyoto Encyclopedia of Genes and Genomes (KEGG) pathway database, Protein Information Resource (PIR) keywords, and InterPro protein database terms. Fellow gene family members of the upstream regulatory candidates were also investigated by review of recent scientific literature.

## Results

### Cross-comparison and regression modeling of PAO levels

**Fig 1:**
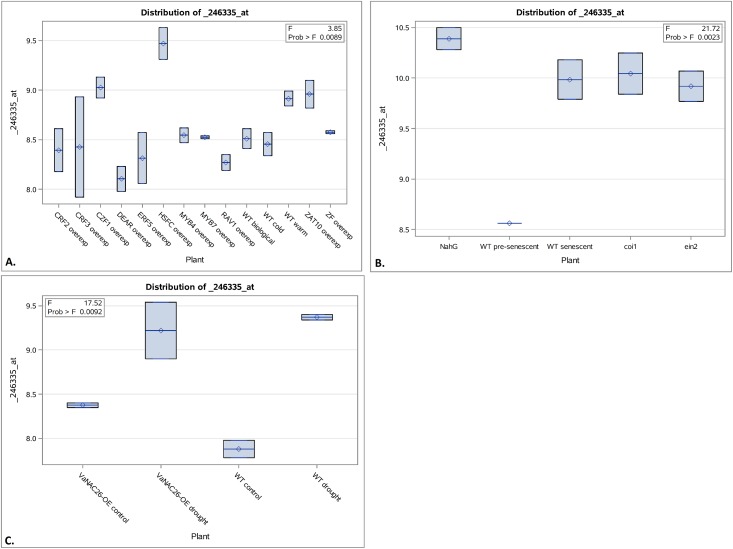
Comparison of PAO expression levels among plant lines in different microarray datasets. An ANOVA was performed to cross-compare PAO expression levels and found significant differences among plant lines in the GSE55907, GSE5727, and GSE72050 microarray datasets at the P<0.05 level in normal vs. pre-freezing treatment (**A**), pre-senescence vs. during senescence (**B**), and drought vs. well-water conditions (**C**), respectively. Box plots displayed lower to upper quartiles with central horizontal lines representing median, diamonds representing mean, end lines representing maximum and minimum values, and circles representing outliers. Further details on the degrees of freedom, mean squares, correlation coefficients, and ANOVA details are provided in S1–S3 Figs.

First, the three microarray datasets were analyzed using ANOVA for comparison of PAO expression levels across all plant lines, environmental conditions, and biological processes, finding in each case that PAO expression levels significantly differed at P<0.05 among the various *Arabidopsis* lines and conditions (Fig 1, S1–S3 Figs) supporting that these datasets would be good sources for finding other genes that were co-regulated with PAO. Next, the microarray datasets were utilized to create multiple linear regression models by automation using both forward and stepwise selection methods at a P = 0.05 cutoff. In particular, there were 24 gene candidates found to predict PAO expression in the GSE55907 dataset as they formed a multiple linear regression model at P<0.05 and R^2^ = 1, receiving the same results with forward and stepwise selection (S1 and S2 Tables). These candidates were then investigated for their functions.

### Functional prediction of gene candidates

The 24 candidates of interest were typified by their function using the SQL query merging the candidate list and pre-loaded ontology data. One candidate, probe name_261564_at, was shown to be a NAC family transcription factor based on SQL query findings (Table 1). The independently conducted DAVID analysis found that TAIR ID AT1G01720 was the only candidate gene with a high probability of being a transcription factor. Both the probe name_261564_at and the TAIR ID AT1G01720 were identified as *Arabidopsis thaliana* Activating Factor 1 (ATAF1). This gene was relevant due to being a transcription factor that was also a member of the NAC domain family, as several other NAC members have also been implicated in the regulation of chlorophyll degradation. thus adding weight to selecting ATAF1 as the most likely candidate for involvement in the PAO regulation (S3 Table).

**Table 1:** Functional descriptions of 24 candidates predicting PAO expression. The 24 candidates predicting PAO levels in the forward and stepwise method multiple linear regression models of the GSE55907 dataset were identified by their functions through performing SQL joins between tables containing gene names and functions. In particular, probe name_261564_at was found to be relevant due to its transcription factor capacity.

Obs	probename	genename	description
**1**	245169_at	AT2G33220	similar to MEE4 (maternal effect embryo arrest 4) [*Arabidopsis* thaliana] (TAIR:AT1G04630.1); similar to F6 [Gossypium hirsutum] (GB:CAC84110.1); contains InterPro domain GRIM-19; (InterPro:IPR009346)
**2**	245604_at	AT4G14290	similar to unknown protein [*Arabidopsis* thaliana] (TAIR:AT3G23540.1); similar to expressed protein [Oryza sativa (japonica cultivar-group)] (GB:ABF96061.1); contains InterPro domain Esterase/lipase/th
**3**	246781_at	AT5G27350	SFP1; carbohydrate transporter/ sugar porter
**4**	247270_at	AT5G64220	calmodulin-binding protein
**5**	247438_at	AT5G62460	zinc finger (C3HC4-type RING finger) family protein
**6**	247792_at	AT5G58787	zinc finger (C3HC4-type RING finger) family protein
**7**	249409_at	AT5G40340	PWWP domain-containing protein
**8**	251208_at	AT3G62880	ATOEP16-4; protein translocase
**9**	251427_at	AT3G60130	glycosyl hydrolase family 1 protein / beta-glucosidase, putative (YLS1)
**10**	251893_at	AT3G54380	SAC3/GANP family protein
**11**	252180_at	AT3G50630	ICK2 (KIP-RELATED PROTEIN 2)
**12**	252570_at	AT3G45300	IVD (ISOVALERYL-COA-DEHYDROGENASE)
**13**	254622_at	AT4G18375	KH domain-containing protein
**14**	255154_at	AT4G08220	
**15**	255386_at	AT4G03620	myosin heavy chain-related
**16**	255535_at	AT4G01790	ribosomal protein L7Ae/L30e/S12e/Gadd45 family protein / ribonuclease P-related
**17**	256084_at	AT1G20750	helicase-related
**18**	256926_at	AT3G22540	similar to unknown protein [*Arabidopsis* thaliana] (TAIR:AT4G14819.1); similar to hypothetical protein MtrDRAFT_AC135320g11v1 [Medicago truncatula] (GB:ABE82963.1); contains InterPro domain Protein of
**19**	261023_at	AT1G12200	flavin-containing monooxygenase family protein / FMO family protein
**20**	261564_at	AT1G01720	ATAF1 (*Arabidopsis* NAC domain containing protein 2); transcription factor
**21**	261880_at	AT1G50500	HIT1 (HEAT-INTOLERANT 1); transporter
**22**	262429_at	AT1G47520	
**23**	264335_s_at	AT1G55860;AT1G70320	[AT1G55860, UPL1 (UBIQUITIN-PROTEIN LIGASE 1); ubiquitin-protein ligase];[AT1G70320, UPL2 (UBIQUITIN-PROTEIN LIGASE 2); ubiquitin-protein ligase]
**24**	265792_at	AT2G01390	pentatricopeptide (PPR) repeat-containing protein

### Correlation and frequency analysis of PAO and gene candidates

**Fig 2:**
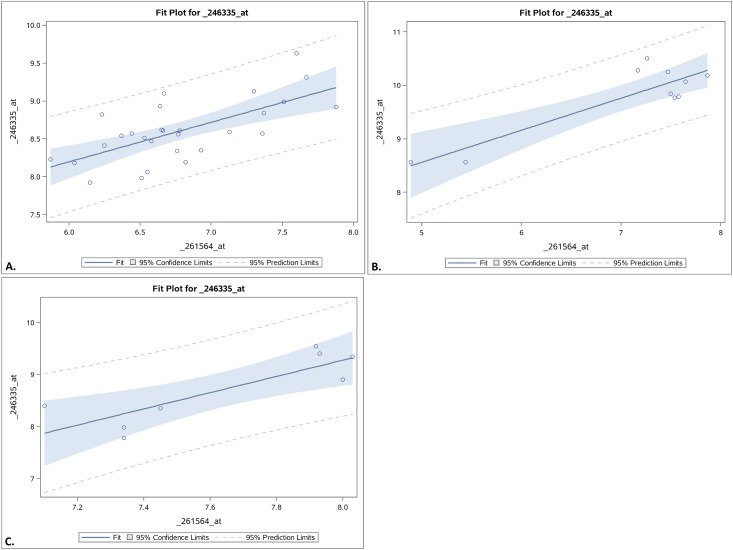
GLM-based fit plot of ATAF1 vs. PAO. The fit plot shows the values of ATAF1 (x-axis) and PAO (y-axis) in each observation, visually showing a positive correlation. The model from the GLM is overlaid onto the existing data of the GSE55907, GSE5727, and GSE72050 microarray datasets and the existing data fit within the 95% prediction limits of the model in normal vs. pre-freezing treatment (**A**), pre-senescence vs. during senescence (**B**), and drought vs. well-water conditions (**C**), respectively.

To investigate ATAF1 further, correlation analysis was performed on ATAF1 compared to PAO, with positive Pearson’s correlation coefficients of greater than 0.5 and P<0.05 being determined between the two variables of ATAF1 and PAO transcript expression levels in all three microarray datasets (Table 2). A GLM was performed using ATAF1 as the sole predictor variable and PAO as the response variable, finding that ATAF1 significantly predicted PAO levels at P<0.05 in all three datasets. These results were overlaid onto a fit plot that contained all observations within 95% prediction limits for all three datasets (Fig 2).

**Table 2:** Correlation analysis of ATAF1 vs. PAO expression. Using Pearson correlation analysis it was found that ATAF1 correlates positively and significantly with PAO at the P<0.05 level in all three microarray datasets. Information about the genetics and physiology of the data sets were also provided.

Data set	Genetics	Physiology	Pearson’s *r*	P value
**GSE55907**	Overexpressing CBF/other TFs	Pre-freezing treatment	0.67205	<0.0001
**GSE5727**	Mutant JA/ethylene and SA pathway	Pre- vs. during senescence	0.88708	0.0006
**GSE72050**	Overexpressing NAC26	Drought vs. well-water	0.84540	0.0082

Finally, frequency analysis repeated for ATAF1 side-by-side with PAO showed that in all three microarray datasets, the two genes correlated positively among plant lines, with ATAF1 levels being lower in plant lines where PAO expression was also low, while ATAF1 levels were higher in plant lines where PAO expression was also high (Table 3). PAO and ATAF1 levels were found to be higher under warmer conditions, during senescence stages, and under drought conditions, as well as in HSFC overexpressing lines, salicylic acid pathway nullifying transgenic line, jasmonic acid/ethylene pathway mutant lines, but were found to be lower in the DEAR overexpressing line. PAO levels were higher but ATAF1 levels slightly lower in NAC26-overexpressing line compared to wild type.

**Table 3:** Frequency analysis of PAO and ATAF1 by plant line. Both PAO (left-side) and ATAF1 (right-side) expression differed by plant line in a positively correlated manner in the GSE55907, GSE5727, and GSE72050 microarray datasets, where PAO and ATAF1 levels were either both high or both low in each plant line. **A.** PAO and ATAF1 levels were higher in warm conditions and certain transcription factor overexpressing lines such as HSFC overexpressing line, and were lower in the DEAR overexpressing line. **B.** PAO and ATAF1 levels were higher in wild-type plants during senescent conditions, in mutant jasmonic acid/ethylene pathway lines, and transgenic salicylic acid pathway line. **C.** PAO and ATAF1 levels were higher in drought conditions in both wild-type and NAC26-overexpressing lines and PAO levels somewhat higher and ATAF1 levels slightly lower in NAC26-overexpressing line compared to wild-type line.

**A**								
	**_246335_at**				**_261564_at**			
	**Mean**	**Std**	**Min**	**Max**	**Mean**	**Std**	**Min**	**Max**
**Plant**	8.40	0.30	8.18	8.61	6.41	0.52	6.04	6.78
**CRF2 overexp**								
**CRF3 overexp**	8.43	0.71	7.92	8.93	6.40	0.35	6.15	6.64
**CZF1 overexp**	9.03	0.15	8.92	9.13	7.59	0.41	7.30	7.88
**DEAR overexp**	8.11	0.18	7.98	8.23	6.19	0.45	5.87	6.51
**ERF5 overexp**	8.32	0.36	8.06	8.57	6.50	0.08	6.44	6.55
**HSFC overexp**	9.47	0.23	9.31	9.63	7.64	0.05	7.60	7.67
**MYB4 overexp**	8.55	0.11	8.47	8.62	6.62	0.05	6.58	6.65
**MYB7 overexp**	8.53	0.02	8.51	8.54	6.45	0.11	6.37	6.53
**RAV1 overexp**	8.27	0.11	8.19	8.35	6.88	0.08	6.82	6.93
**WT biological**	8.51	0.14	8.41	8.61	6.46	0.29	6.25	6.66
**WT cold**	8.46	0.16	8.34	8.57	7.06	0.42	6.76	7.36
**WT warm**	8.92	0.11	8.84	8.99	7.44	0.10	7.37	7.51
**ZAT10 overexp**	8.96	0.20	8.82	9.10	6.45	0.31	6.23	6.67
**ZF overexp**	8.58	0.02	8.56	8.59	6.95	0.25	6.77	7.13
**B**								
	**_246335_at**				**_261564_at**			
	**Mean**	**Std**	**Min**	**Max**	**Mean**	**Std**	**Min**	**Max**
**Plant**	10.39	0.16	10.28	10.50	7.22	0.06	7.17	7.26
**NahG**								
**WT pre-senescent**	8.56	0.00	8.56	8.56	5.17	0.39	4.89	5.44
**WT senescent**	9.99	0.28	9.79	10.18	7.73	0.21	7.58	7.87
**coi1**	10.05	0.29	9.84	10.25	7.49	0.02	7.47	7.50
**ein2**	9.92	0.21	9.77	10.07	7.60	0.08	7.54	7.65
**C**								
	**_246335_at**				**_261564_at**			
	**Mean**	**Std**	**Min**	**Max**	**Mean**	**Std**	**Min**	**Max**
**Plant**	8.38	0.04	8.35	8.40	7.28	0.25	7.10	7.45
**VaNAC26-OE control**								
**VaNAC26-OE drought**	9.22	0.45	8.90	9.54	7.96	0.06	7.92	8.00
**WT control**	7.88	0.14	7.78	7.98	7.34	0.00	7.34	7.34
**WT drought**	9.37	0.04	9.34	9.40	7.98	0.07	7.93	8.03

## Discussion

The analysis of the GSE55907, GSE5727, and GSE72050 microarray datasets revealed that PAO expression differed among the different plant lines, which were either wild type, overexpressing, transgenic, or mutant lines, as well as among the differing experimental conditions such as pre-freezing treatment vs. normal conditions, before vs. during senescence, or drought vs. well-water conditions (Fig 1, S1–S3 Figs). These analyses indicated that both the genetic background as well as environmental conditions needed to be taken into account when analyzing PAO and its regulators.

To determine the potential indirect genetic factors regulating PAO expression in *Arabidopsis* in particular, the list of total genes in *Arabidopsis* was narrowed down by whether there was a significant correlation with P<0.05 and then narrowed down further by whether the genes would predict PAO, resulting in 24 gene candidates, one of which was the transcription factor ATAF1 (Table 1, S1–S3 Tables). This is relevant because PAO is known to be transcriptionally regulated [4], and ATAF1, a NAC domain family transcription factor, has been found in the analyses presented to be involved with PAO expression across wild type and transgenic plant lines, in freezing, senescence, and drought experimental microarray datasets.

**Fig 3:**
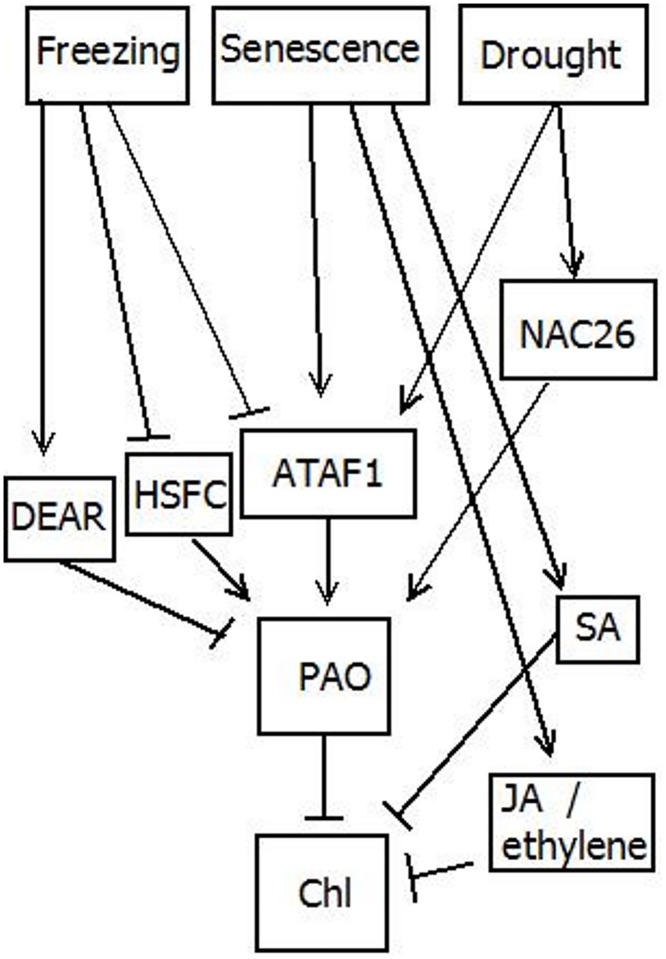
Schematic of regulatory relationship between various abiotic and biotic factors, ATAF1, PAO, DEAR, HSFC, and NAC26 expression, jasmonic acid (JA)/ethylene pathways, salicylic acid (SA) pathway, and chlorophyll (Chl). The above schematic presents a summary of the findings and our current understanding of the relationship between the three different conditions, cold, senescence, and drought, with ATAF1 and PAO gene expression and chlorophyll (Chl) levels in plants. Pointed arrows represent direct relationships between the entities while blind-ended arrows represent inverse relationships between the entities.

Further correlation analysis and regression modeling pointed to the same conclusion that ATAF1 significantly predicts PAO and that PAO is positively correlated with ATAF1 (Table 2, Fig 2). Frequency analysis showed that PAO and ATAF1 expression levels were either both high or both low in the various plant lines, again reinforcing the positive correlation between ATAF1 and PAO (Table 3). In particular, PAO and ATAF1 were both overexpressed under warmer conditions, during senescence, and under drought conditions as well as in certain plant lines like HSFC overexpressing lines. PAO and ATAF1 were also overexpressed in jasmonic acid/ethylene pathway mutant lines and salicylic acid pathway transgenic line despite pathway disruption in these lines, suggesting that jasmonic acid/ethylene pathway and salicylic acid pathway, which are involved in senescence-induction, work independently of ATAF1 to induce chlorophyll degradation. PAO levels were also higher despite slightly lower ATAF1 levels in the NAC26-overexpressing line, suggesting that NAC26 up-regulates PAO in a manner similar to ATAF1. Meanwhile both PAO and ATAF1 were underexpressed in the DEAR overexpressing line. These findings are compiled into a working model showing hypothesized relationships among the different elements that interact to regulate PAO expression (Fig 3).

Our model is further supported by several findings in recent literature showing a strong connection between NAC family domain transcription factors and various abiotic factors and plant processes, including temperature and leaf senescence, serving as important regulators. For example, NAC domain family member NAM-B1 was found to accelerate senescence and increase zinc and iron content in wheat grain [23], while another member JUNGBRUNNEN1 was found to regulate longevity and tolerance to abiotic stress including salt, cold, and heat in *Arabidopsis* plants [24] and NAC family member AtNAP was found to trigger senescence when overexpressed [25]. These findings suggest that ATAF1 plays similar roles in pre-freezing treatment, senescence, and drought conditions, likely due to their similar protein composition containing the same NAC domain. Recent literature suggests a similar scheme for ethylene signaling and leaf senescence-induced chlorophyll degradation through ethylene insensitive 3 (EIN3), similar to ein2 gene investigated here, acting through another NAC domain gene ORE1, similar to ATAF1 gene, to modulate PAO expression [26]. ATAF1 overexpressing lines were also found to have stunted growth and delayed flowering [27], affirming connections with chlorophyll degradation.

## Conclusion

PAO transcript expression was found to be significantly up-regulated in warm conditions, during leaf senescence, and in drought conditions, and in all three conditions significantly positively correlated with expression of ATAF1, a NAC transcription factor implicated in the literature as being related to all three of these types of conditions. This analysis posits a regulatory network in which ATAF1 is triggered in response to these abiotic stresses and acts to regulate chlorophyll degradation by up-regulating PAO expression.

## Supporting Information

S1 Fig ANOVA of PAO expression among plant lines in GSE55907 dataset. PAO is represented by probe name _246335_at, while Plant class variable represents the 14 plant lines of the study representing wild type under different temperatures, or transgenic lines overexpressing CBF-induced transcription factors. Note significant factor of Plant (p = 0.0089). Box plots displayed lower to upper quartiles with central horizontal line representing median, and diamond representing mean.
(TIF)

S2 Fig ANOVA of PAO expression among plant lines in GSE5727 dataset. PAO is represented by probe name _246335_at, while Plant class variable represents the 5 plant lines of the study representing wild type pre- and during senescence, or mutant or transgenic lines, in relevant senescence pathways. Note significant factor of Plant (p = 0.0023). Box plots displayed lower to upper quartiles with central horizontal line representing median, and diamond representing mean.
(TIF)

S3 Fig ANOVA of PAO expression among plant lines in GSE72050 dataset. PAO is represented by probe name _246335_at, while Plant class variable represents the 4 plant lines of the study representing wild-type or NAC26 line in well-water or drought conditions. Note significant factor of Plant (p = 0.0092). Box plots displayed lower to upper quartiles with central horizontal line representing median, and diamond representing mean.
(TIF)

S1 Table Stepwise multiple linear regression to predict PAO through 24 correlated genes. After performing stepwise selection with a multiple linear regression model containing all PAO-correlated genes, these 24 candidates were identified as together forming a significant model (p<0.05) predicting PAO expression and explaining all variation in PAO expression (R^2^ = 1).
(DOCX)

S2 Table Forward multiple linear regression to predict PAO through 24 correlated genes. After performing forward selection with a multiple linear regression model containing all PAO-correlated genes, these 24 candidates were identified as together forming a significant model (p<0.05) predicting PAO expression and explaining all variation in PAO expression (R^2^ = 1).
(DOCX)

S3 Table Further functional descriptions of 24 candidates predicting PAO expression. The 24 candidates predicting PAO in the multiple linear regression models were annotated using the DAVID tool. In particular, probe name_261564_at, here aliased by TAIR ID AT1G01720, was found to be relevant due to its transcription factor capacity.
(DOCX)

S1 Appendix SAS code for statistical analysis of microarray datasets. The written SAS code is presented below for the statistical analysis of the GSE55907, GSE5727, and GSE72050 microarray datasets.
(DOCX)
