# Supplementary figures and images for: Investigating the Control of Chlorophyll Degradation by Genomic Correlation Mining
Source: PLoS One. 2016 Sep 12;11(9):e0162327. doi: 10.1371/journal.pone.0162327 (PMC5019398; doi:10.1371/journal.pone.0162327)

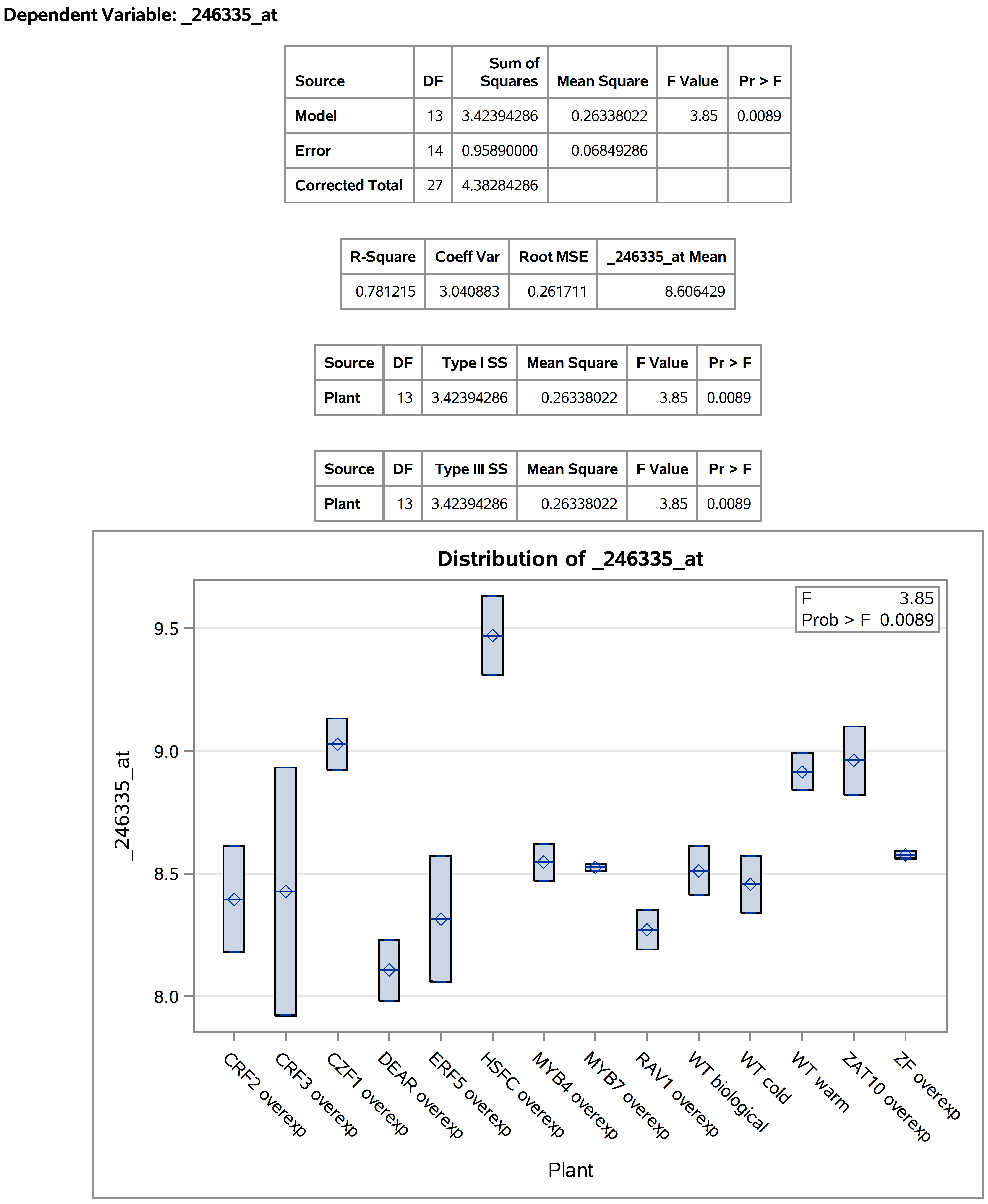

Supplement: S1 Fig — PAO is represented by probe name _246335_at, while Plant class variable represents the 14 plant lines of the study representing wild type under different temperatures, or transgenic lines overexpressing CBF-induced transcription factors. Note significant factor of Plant (p = 0.0089). Box plots displayed lower to upper quartiles with central horizontal line representing median, and diamond representing mean. (TIF) [file pone.0162327.s001.tif]

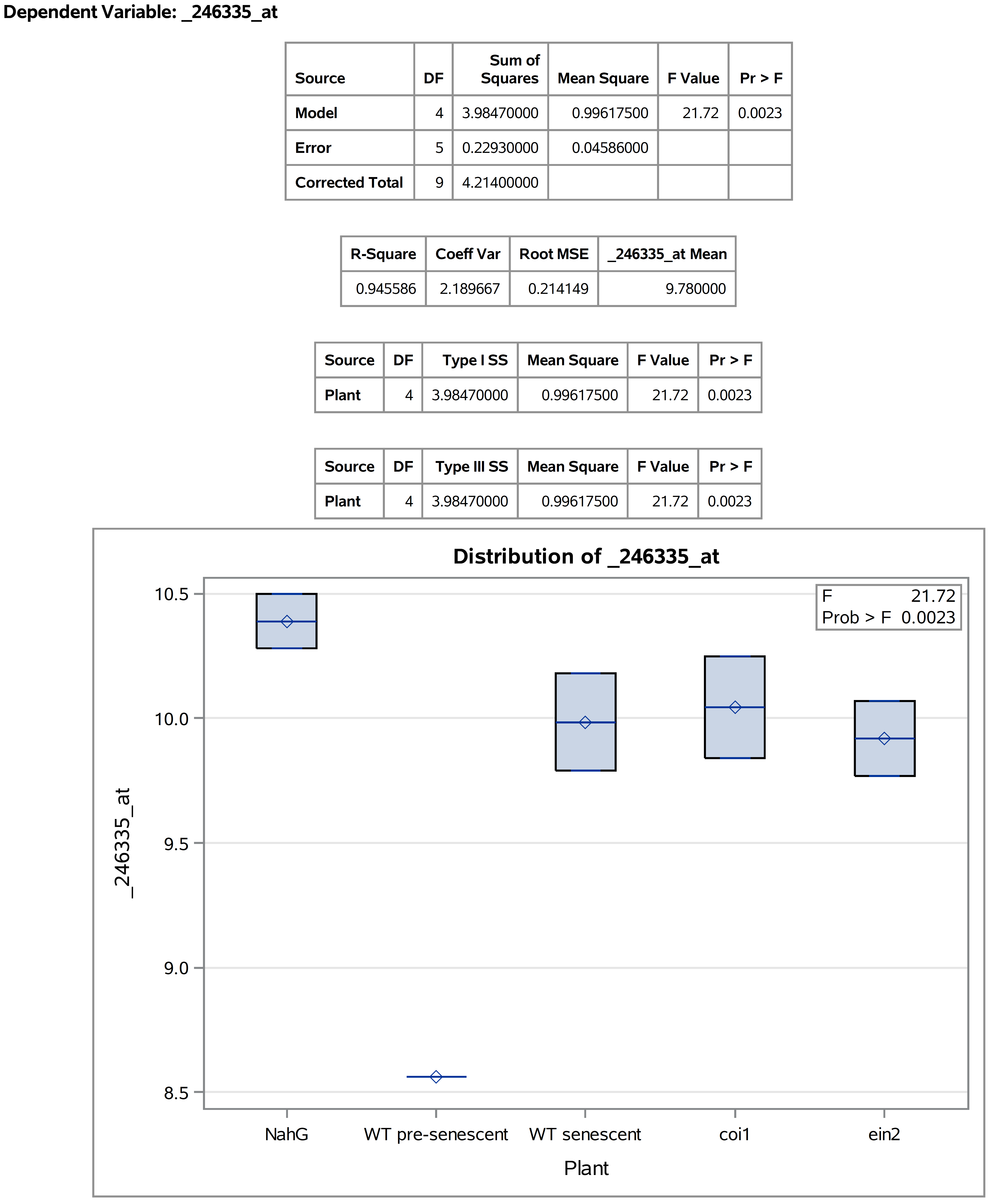

Supplement: S2 Fig — PAO is represented by probe name _246335_at, while Plant class variable represents the 5 plant lines of the study representing wild type pre- and during senescence, or mutant or transgenic lines, in relevant senescence pathways. Note significant factor of Plant (p = 0.0023). Box plots displayed lower to upper quartiles with central horizontal line representing median, and diamond representing mean. (TIF) [file pone.0162327.s002.tif]

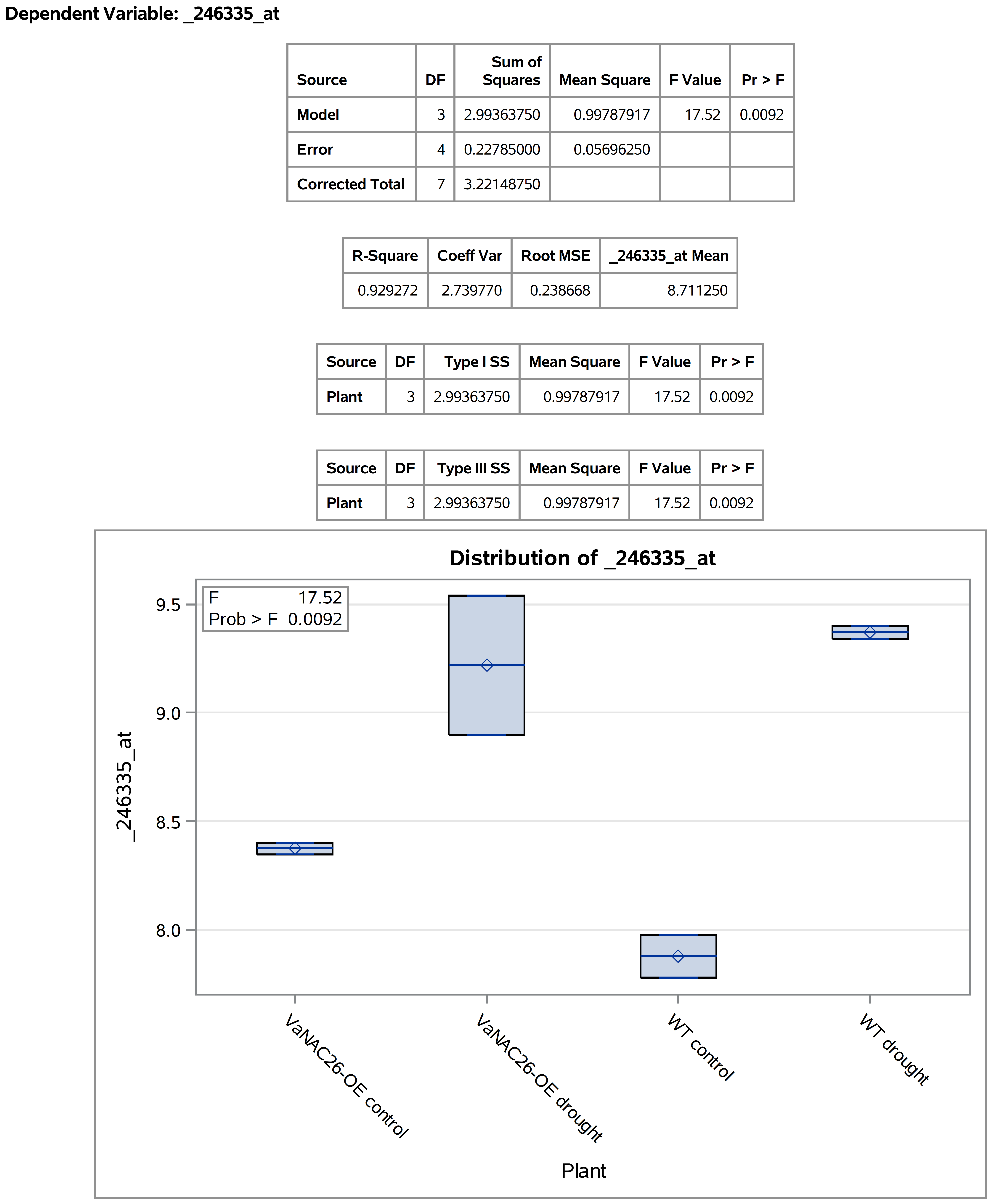

Supplement: S3 Fig — PAO is represented by probe name _246335_at, while Plant class variable represents the 4 plant lines of the study representing wild-type or NAC26 line in well-water or drought conditions. Note significant factor of Plant (p = 0.0092). Box plots displayed lower to upper quartiles with central horizontal line representing median, and diamond representing mean. (TIF) [file pone.0162327.s003.tif]
