# Supplementary material for: Investigating the Control of Chlorophyll Degradation by Genomic Correlation Mining
Source: PLoS One. 2016 Sep 12;11(9):e0162327. doi: 10.1371/journal.pone.0162327 (PMC5019398; doi:10.1371/journal.pone.0162327)
Supplement: S2 Table — After performing forward selection with a multiple linear regression model containing all PAO-correlated genes, these 24 candidates were identified as together forming a significant model (p<0.05) predicting PAO expression and explaining all variation in PAO expression (R2 = 1). (DOCX) [file pone.0162327.s005.docx]

**S2 Table. Forward multiple linear regression to predict PAO through 24 correlated genes.** After performing forward selection with a multiple linear regression model containing all PAO-correlated genes, these 24 candidates were identified as together forming a significant model (p<0.05) predicting PAO expression and explaining all variation in PAO expression (R^2^ = 1).

| **Summary of Forward Selection** | | | | | | | |
| --- | --- | --- | --- | --- | --- | --- | --- |
| **Step** | **Variable Entered** | **Number Vars In** | **Partial R-Square** | **Model R-Square** | **C(p)** | **F Value** | **Pr > F** |
| **1** | _247792_at | 1 | 0.6675 | 0.6675 | . | 52.19 | <.0001 |
| **2** | _251208_at | 2 | 0.1708 | 0.8383 | . | 26.41 | <.0001 |
| **3** | _247438_at | 3 | 0.0715 | 0.9098 | . | 19.02 | 0.0002 |
| **4** | _261023_at | 4 | 0.0455 | 0.9553 | . | 23.45 | <.0001 |
| **5** | _256926_at | 5 | 0.0212 | 0.9766 | . | 19.96 | 0.0002 |
| **6** | _265792_at | 6 | 0.0066 | 0.9832 | . | 8.27 | 0.0090 |
| **7** | _255386_at | 7 | 0.0074 | 0.9906 | . | 15.80 | 0.0007 |
| **8** | _249409_at | 8 | 0.0043 | 0.9949 | . | 16.24 | 0.0007 |
| **9** | _251427_at | 9 | 0.0031 | 0.9981 | . | 29.62 | <.0001 |
| **10** | _252570_at | 10 | 0.0012 | 0.9993 | . | 27.08 | <.0001 |
| **11** | _251893_at | 11 | 0.0004 | 0.9997 | . | 23.01 | 0.0002 |
| **12** | _261880_at | 12 | 0.0001 | 0.9998 | . | 11.16 | 0.0045 |
| **13** | _254622_at | 13 | 0.0001 | 0.9999 | . | 12.61 | 0.0032 |
| **14** | _245169_at | 14 | 0.0001 | 1.0000 | . | 19.23 | 0.0007 |
| **15** | _247270_at | 15 | 0.0000 | 1.0000 | . | 32.57 | <.0001 |
| **16** | _256084_at | 16 | 0.0000 | 1.0000 | . | 71.57 | <.0001 |
| **17** | _246781_at | 17 | 0.0000 | 1.0000 | . | 43.08 | <.0001 |
| **18** | _245604_at | 18 | 0.0000 | 1.0000 | . | 40.63 | 0.0001 |
| **19** | _252180_at | 19 | 0.0000 | 1.0000 | . | 32.87 | 0.0004 |
| **20** | _255535_at | 20 | 0.0000 | 1.0000 | . | 40.49 | 0.0004 |
| **21** | _261564_at | 21 | 0.0000 | 1.0000 | . | 29.26 | 0.0016 |
| **22** | _262429_at | 22 | 0.0000 | 1.0000 | . | 55.72 | 0.0007 |
| **23** | _255154_at | 23 | 0.0000 | 1.0000 | . | Infty | <.0001 |
| **24** | _264335_s_at | 24 | 0.0000 | 1.0000 | . | Infty | <.0001 |
