# Supplementary material for: Investigating the Control of Chlorophyll Degradation by Genomic Correlation Mining
Source: PLoS One. 2016 Sep 12;11(9):e0162327. doi: 10.1371/journal.pone.0162327 (PMC5019398; doi:10.1371/journal.pone.0162327)
Supplement: S3 Table — The 24 candidates predicting PAO in the multiple linear regression models were annotated using the DAVID tool. In particular, probe name_261564_at, here aliased by TAIR ID AT1G01720, was found to be relevant due to its transcription factor capacity. (DOCX) [file pone.0162327.s006.docx]

**S3 Table. Further functional descriptions of 24 candidates predicting PAO expression.** The 24 candidates predicting PAO in the multiple linear regression models were annotated using the DAVID tool. In particular, probe name_261564_at, here aliased by TAIR ID AT1G01720, was found to be relevant due to its transcription factor capacity.

| AT1G12200 | AT1G12200 | Related Genes | *Arabidopsis* thaliana |
| --- | --- | --- | --- |
| GOTERM_MF_FAT | nucleotide binding, nucleoside binding, purine nucleoside binding, flavin-containing monooxygenase activity, purine nucleotide binding, adenyl nucleotide binding, cofactor binding, FAD binding, NADP or NADPH binding, coenzyme binding, | | |
| INTERPRO | Flavin-containing monooxygenase FMO, | | |
| SP_PIR_KEYWORDS | complete proteome, | | |
| AT1G20750 | AT1G20750 | Related Genes | *Arabidopsis* thaliana |
| GOTERM_CC_FAT | chloroplast, plastid, | | |
| GOTERM_MF_FAT | nucleotide binding, nucleoside binding, purine nucleoside binding, DNA binding, DNA helicase activity, ATP-dependent DNA helicase activity, helicase activity, ATP binding, ATP-dependent helicase activity, DNA-dependent ATPase activity, ATPase activity, purine nucleotide binding, adenyl nucleotide binding, ribonucleotide binding, purine ribonucleotide binding, adenyl ribonucleotide binding, ATPase activity, coupled, purine NTP-dependent helicase activity, | | |
| AT1G50500 | AT1G50500 | Related Genes | *Arabidopsis* thaliana |
| GOTERM_BP_FAT | retrograde vesicle-mediated transport, Golgi to ER, response to osmotic stress, response to temperature stimulus, response to heat, response to abiotic stimulus, vesicle-mediated transport, intracellular transport, Golgi vesicle transport, | | |
| GOTERM_CC_FAT | mitochondrion, | | |
| INTERPRO | Vps53-like, N-terminal, | | |
| SP_PIR_KEYWORDS | complete proteome, | | |
| AT2G33220 | AT2G33220 | Related Genes | *Arabidopsis* thaliana |
| GOTERM_BP_FAT | photorespiration, cellular metabolic compound salvage, | | |
| GOTERM_CC_FAT | mitochondrion, mitochondrial envelope, plastid, NADH dehydrogenase complex, organelle membrane, mitochondrial membrane, organelle envelope, envelope, mitochondrial part, respiratory chain complex I, respiratory chain, | | |
| INTERPRO | GRIM-19, | | |
| KEGG_PATHWAY | Oxidative phosphorylation, | | |
| SP_PIR_KEYWORDS | complete proteome, | | |
| AT3G22540 | AT3G22540 | Related Genes | *Arabidopsis* thaliana |
| INTERPRO | Protein of unknown function DUF1677, plant, | | |
| SP_PIR_KEYWORDS | complete proteome, | | |
| AT3G54380 | AT3G54380 | Related Genes | *Arabidopsis* thaliana |
| INTERPRO | SAC3/GANP/Nin1/mts3/eIF-3 p25, | | |
| SP_PIR_KEYWORDS | complete proteome, | | |
| AT3G62880 | AT3G62880 | Related Genes | *Arabidopsis* thaliana |
| GOTERM_BP_FAT | protein localization, protein transport, establishment of protein localization, | | |
| GOTERM_CC_FAT | mitochondrion, mitochondrial envelope, mitochondrial inner membrane, mitochondrial inner membrane presequence translocase complex, plastid envelope, plastid outer membrane, plastid, organelle inner membrane, outer membrane, organelle membrane, mitochondrial membrane, organelle envelope, organelle outer membrane, envelope, plastid membrane, mitochondrial part, plastid part, mitochondrial membrane part, | | |
| GOTERM_MF_FAT | protein transmembrane transporter activity, protein transporter activity, primary active transmembrane transporter activity, P-P-bond-hydrolysis-driven transmembrane transporter activity, P-P-bond-hydrolysis-driven protein transmembrane transporter activity, macromolecule transmembrane transporter activity, | | |
| INTERPRO | Mitochondrial inner membrane translocase complex, subunit Tim17/22, | | |
| SP_PIR_KEYWORDS | complete proteome, | | |
| AT4G01790 | AT4G01790 | Related Genes | *Arabidopsis* thaliana |
| GOTERM_MF_FAT | nuclease activity, endonuclease activity, endoribonuclease activity, ribonuclease P activity, ribonuclease activity, tRNA-specific ribonuclease activity, endoribonuclease activity, producing 5'-phosphomonoesters, endonuclease activity, active with either ribo- or deoxyribonucleic acids and producing 5'-phosphomonoesters, | | |
| INTERPRO | Ribosomal protein L7Ae/L30e/S12e/Gadd45, | | |
| SP_PIR_KEYWORDS | complete proteome, | | |
| AT4G03620 | AT4G03620 | Related Genes | *Arabidopsis* thaliana |
| SP_PIR_KEYWORDS | complete proteome, | | |
| AT4G14290 | AT4G14290 | Related Genes | *Arabidopsis* thaliana |
| GOTERM_CC_FAT | plasma membrane, | | |
| AT5G40340 | AT5G40340 | Related Genes | *Arabidopsis* thaliana |
| GOTERM_CC_FAT | nucleolus, membrane-enclosed lumen, nuclear lumen, non-membrane-bounded organelle, intracellular non-membrane-bounded organelle, organelle lumen, intracellular organelle lumen, | | |
| INTERPRO | PWWP, | | |
| SMART | PWWP, | | |
| SP_PIR_KEYWORDS | complete proteome, | | |
| AT5G58787 | AT5G58787 | Related Genes | *Arabidopsis* thaliana |
| GOTERM_MF_FAT | zinc ion binding, ion binding, cation binding, metal ion binding, transition metal ion binding, | | |
| INTERPRO | Zinc finger, RING-type, Zinc finger, RING-type, conserved site, Zinc finger, C3HC4 RING-type, | | |
| SMART | RING, | | |
| SP_PIR_KEYWORDS | complete proteome, metal-binding, zinc, zinc-finger, | | |
| AT5G62460 | AT5G62460 | Related Genes | *Arabidopsis* thaliana |
| GOTERM_MF_FAT | zinc ion binding, ion binding, cation binding, metal ion binding, transition metal ion binding, | | |
| INTERPRO | Zinc finger, RING-CH-type, Zinc finger, C3HC4 RING-type, | | |
| PIR_SUPERFAMILY | PIRSF026887:PIRSF026887, | | |
| SMART | RINGv, | | |
| SP_PIR_KEYWORDS | complete proteome, | | |
| AT3G60130 | Beta-galactosidase 16 | Related Genes | *Arabidopsis* thaliana |
| GOTERM_CC_FAT | extracellular region, endomembrane system, apoplast, | | |
| GOTERM_MF_FAT | beta-galactosidase activity, sugar binding, galactosidase activity, carbohydrate binding, ion binding, cation binding, | | |
| INTERPRO | D-galactoside/L-rhamnose binding SUEL lectin, Glycoside hydrolase, family 1, Glycoside hydrolase, family 35, Glycoside hydrolase, subgroup, catalytic core, Glycoside hydrolase, family 35, conserved site, Legume lectin, beta chain, Mn/Ca-binding site, | | |
| SP_PIR_KEYWORDS | alternative splicing, complete proteome, disulfide bond, glycoprotein, glycosidase, hydrolase, signal, | | |
| UP_SEQ_FEATURE | active site:Nucleophile, active site:Proton donor, binding site:Substrate, chain:Beta-glucosidase 16, disulfide bond, glycosylation site:N-linked (GlcNAc...), region of interest:Substrate binding, signal peptide, splice variant, | | |
| AT5G64220 | Calmodulin-binding transcription activator 2 | Related Genes | *Arabidopsis* thaliana |
| GOTERM_BP_FAT | transcription, regulation of transcription, | | |
| GOTERM_MF_FAT | DNA binding, calcium ion binding, calmodulin binding, transcription regulator activity, ion binding, cation binding, metal ion binding, | | |
| INTERPRO | IQ calmodulin-binding region, Ankyrin, CG-1, | | |
| SMART | IQ, ANK, | | |
| SP_PIR_KEYWORDS | activator, ank repeat, calcium, calmodulin-binding, coiled coil, complete proteome, dna-binding, nucleus, phosphoprotein, repeat, stress response, Transcription, transcription regulation, | | |
| UP_SEQ_FEATURE | chain:Calmodulin-binding transcription activator 2, compositionally biased region:Poly-Ser, DNA-binding region:CG-1, domain:IQ 1, domain:IQ 2, modified residue, repeat:ANK 1, repeat:ANK 2, | | |
| AT3G50630 | Cyclin-dependent kinase inhibitor 2 | Related Genes | *Arabidopsis* thaliana |
| GOTERM_BP_FAT | regulation of cyclin-dependent protein kinase activity, DNA metabolic process, DNA replication, DNA-dependent DNA replication, negative regulation of protein kinase activity, cell cycle, cell cycle arrest, regulation of phosphate metabolic process, cell cycle process, negative regulation of kinase activity, DNA endoreduplication, regulation of phosphorylation, negative regulation of catalytic activity, regulation of kinase activity, negative regulation of molecular function, negative regulation of cyclin-dependent protein kinase activity, negative regulation of cell cycle, regulation of protein kinase activity, regulation of phosphorus metabolic process, regulation of transferase activity, negative regulation of transferase activity, regulation of cell cycle, | | |
| GOTERM_CC_FAT | nucleoplasm, membrane-enclosed lumen, nuclear lumen, organelle lumen, intracellular organelle lumen, | | |
| GOTERM_MF_FAT | enzyme inhibitor activity, protein kinase inhibitor activity, cyclin-dependent protein kinase inhibitor activity, cyclin-dependent protein kinase regulator activity, kinase regulator activity, kinase inhibitor activity, protein kinase regulator activity, protein serine/threonine kinase inhibitor activity, | | |
| INTERPRO | Cyclin-dependent kinase inhibitor, Cyclin-dependent kinase inhibitor, plant, | | |
| PIR_SUPERFAMILY | PIRSF017811:CDK_inhib_pln, PIRSF017811:cyclin-dependent kinase inhibitor protein, plant type, | | |
| SP_PIR_KEYWORDS | cell cycle, complete proteome, nucleus, phosphoprotein, protein kinase inhibitor, | | |
| UP_SEQ_FEATURE | chain:Cyclin-dependent kinase inhibitor 2, region of interest:Required for nuclear localization, | | |
| AT3G45300 | Isovaleryl-CoA dehydrogenase, mitochondrial | Related Genes | *Arabidopsis* thaliana |
| COG_ONTOLOGY | Lipid metabolism, | | |
| GOTERM_BP_FAT | oxidation reduction, | | |
| GOTERM_CC_FAT | mitochondrion, mitochondrial matrix, membrane-enclosed lumen, mitochondrial lumen, organelle lumen, mitochondrial part, intracellular organelle lumen, | | |
| GOTERM_MF_FAT | nucleotide binding, nucleoside binding, purine nucleoside binding, acyl-CoA dehydrogenase activity, ATP binding, isovaleryl-CoA dehydrogenase activity, electron carrier activity, purine nucleotide binding, adenyl nucleotide binding, ribonucleotide binding, purine ribonucleotide binding, adenyl ribonucleotide binding, cofactor binding, FAD binding, coenzyme binding, | | |
| INTERPRO | Acyl-CoA dehydrogenase, conserved site, Acyl-CoA oxidase/dehydrogenase, type 1, Acyl-CoA oxidase/dehydrogenase, central region, Acyl-CoA dehydrogenase, N-terminal, Acyl-CoA oxidase/dehydrogenase, type1/2, C-terminal, Acyl-CoA dehydrogenase/oxidase, N-terminal, | | |
| KEGG_PATHWAY | Valine, leucine and isoleucine degradation, | | |
| SP_PIR_KEYWORDS | complete proteome, FAD, Flavoprotein, mitochondrion, oxidoreductase, transit peptide, | | |
| UP_SEQ_FEATURE | active site:Proton acceptor, binding site:FAD, binding site:Substrate, binding site:Substrate; via carbonyl oxygen, chain:Isovaleryl-CoA dehydrogenase, mitochondrial, nucleotide phosphate-binding region:FAD, region of interest:Substrate binding, sequence conflict, transit peptide:Mitochondrion, | | |
| AT4G18375 | KH domain-containing protein At4g18375 | Related Genes | *Arabidopsis* thaliana |
| GOTERM_MF_FAT | RNA binding, | | |
| INTERPRO | K Homology, K Homology, type 1, K Homology, type 1, subgroup, | | |
| SMART | KH, | | |
| SP_PIR_KEYWORDS | alternative splicing, complete proteome, nucleus, repeat, rna-binding, | | |
| UP_SEQ_FEATURE | chain:KH domain-containing protein At4g18375, domain:KH 1, domain:KH 2, domain:KH 3, domain:KH 4, domain:KH 5, splice variant, | | |
| AT1G01720 | NAC domain-containing protein 2 | Related Genes | *Arabidopsis* thaliana |
| GOTERM_BP_FAT | transcription, response to wounding, regulation of abscisic acid mediated signaling, negative regulation of abscisic acid mediated signaling, negative regulation of signal transduction, negative regulation of cell communication, regulation of transcription, negative regulation of response to stimulus, | | |
| GOTERM_MF_FAT | DNA binding, transcription factor activity, transcription activator activity, transcription regulator activity, | | |
| INTERPRO | No apical meristem (NAM) protein, | | |
| SP_PIR_KEYWORDS | complete proteome, dna-binding, nucleus, Transcription, transcription regulation, | | |
| UP_SEQ_FEATURE | chain:NAC domain-containing protein 2, domain:NAC, | | |
| AT2G01390 | Pentatricopeptide repeat-containing protein At2g01390 | Related Genes | *Arabidopsis* thaliana |
| GOTERM_CC_FAT | chloroplast, plastid, | | |
| INTERPRO | Pentatricopeptide repeat, | | |
| SP_PIR_KEYWORDS | complete proteome, repeat, | | |
| UP_SEQ_FEATURE | chain:Pentatricopeptide repeat-containing protein At2g01390, repeat:PPR 1, repeat:PPR 10, repeat:PPR 2, repeat:PPR 3, repeat:PPR 4, repeat:PPR 5, repeat:PPR 6, repeat:PPR 7, repeat:PPR 8, repeat:PPR 9, sequence conflict, | | |
| AT5G27350 | Sugar transporter ERD6-like 17 | Related Genes | *Arabidopsis* thaliana |
| GOTERM_BP_FAT | carbohydrate transport, response to nematode, transmembrane transport, | | |
| GOTERM_CC_FAT | integral to membrane, intrinsic to membrane, | | |
| GOTERM_MF_FAT | sugar:hydrogen symporter activity, cation:sugar symporter activity, symporter activity, solute:cation symporter activity, solute:hydrogen symporter activity, sugar transmembrane transporter activity, | | |
| INTERPRO | Sugar/inositol transporter, General substrate transporter, Sugar transporter, conserved site, | | |
| PIR_SUPERFAMILY | PIRSF005322:glucose transport protein, | | |
| SP_PIR_KEYWORDS | complete proteome, membrane, sugar transport, transmembrane, transport, | | |
| UP_SEQ_FEATURE | chain:Sugar transporter ERD6-like 17, sequence conflict, transmembrane region, | | |
