# Supplementary material for: Investigating the Control of Chlorophyll Degradation by Genomic Correlation Mining
Source: PLoS One. 2016 Sep 12;11(9):e0162327. doi: 10.1371/journal.pone.0162327 (PMC5019398; doi:10.1371/journal.pone.0162327)
Supplement: S1 Appendix — The written SAS code is presented below for the statistical analysis of the GSE55907, GSE5727, and GSE72050 microarray datasets. (DOCX) [file pone.0162327.s007.docx]

**S1 Appendix. SAS code for statistical analysis of microarray datasets.** The written SAS code is presented below for the statistical analysis of the GSE55907, GSE5727, and GSE72050 microarray datasets.

**GSE55907 Code**

options nodate nonumber;

ods pdf file= 'gse55907microarray.pdf';

libname micro 'c:\labwork';

/*microarray import*/

proc import datafile='GSE55907.csv' out=micro.gds416 dbms=csv replace;

getnames=yes;

guessingrows=32767; /*to avoid data truncation*/

run;

proc transpose data=micro.gds416 out=micro.gds416trans;

id _;

run;

/*Affy probe names*/

data affy2010 (keep = probename genename description);

infile 'affy2010probenames.txt' dlm='09'x dsd firstobs=2;

length probename $40 x1 $100 x2 $100 x3 $100 genename $40 description $200;

input probename $ x1 $ x2 $ x3 $ genename $ description $;

run;

/*proc print data=affy2010;

where genename= 'AT3G44880'; PAO gene code

run;

var _246335_at probe name is our PAO*/

data micro.gds416transmod;

set micro.gds416trans;

length Plant $20;

Plant1 = input(compress(_NAME_, '_'),best12.);

if 1348266 <= Plant1 < 1348268 then Plant = 'WT warm';

else if 1348268<= Plant1 < 1348270 then Plant = 'WT cold';

else if 1348270<= Plant1 < 1348272 then Plant = 'WT biological';

else if 1348272<= Plant1 < 1348274 then Plant = 'CRF2 overexp';

else if 1348274<= Plant1 < 1348276 then Plant = 'CRF3 overexp';

else if 1348276<= Plant1 < 1348278 then Plant = 'CZF1 overexp';

else if 1348278<= Plant1 < 1348280 then Plant = 'DEAR overexp';

else if 1348280<= Plant1 < 1348282 then Plant = 'ERF5 overexp';

else if 1348282<= Plant1 < 1348284 then Plant = 'HSFC overexp';

else if 1348284<= Plant1 < 1348286 then Plant = 'MYB4 overexp';

else if 1348286<= Plant1 < 1348288 then Plant = 'MYB7 overexp';

else if 1348288<= Plant1 < 1348290 then Plant = 'RAV1 overexp';

else if 1348290<= Plant1 < 1348292 then Plant = 'ZAT10 overexp';

else if 1348292<= Plant1 < 1348294 then Plant = 'ZF overexp';

drop Plant1;

run;

title 'Experimental Plant Frequencies';

proc freq data=micro.gds416transmod nlevels;

tables Plant;

run;

title 'PAO expression stats by Plant';

proc tabulate data=micro.gds416transmod;

class Plant;

var _246335_at;

tables Plant,_246335_at*(MEAN STD MIN MAX);

run;

/*PAO analysis by Plant*/

title 'Analysis of Variance (ANOVA) of PAO based on Plant';

proc glm data=micro.gds416transmod;

class Plant;

model _246335_at= Plant;

means Plant /tukey hovtest=BF;

lsmeans Plant;

output out=diagnostics p=ybar r=resid cookd=CookD;

run;

title2 'Diagnostics for ANOVA';

proc univariate data=diagnostics normal;

var resid;

qqplot /normal;

run;

proc sort data=micro.gds416transmod;

by Plant;

run;

/*Correlations*/

title 'Pearson Correlation for PAO vs. other genes';

proc corr data=micro.gds416transmod pearson nosimple noprint rank outp=correlations;

var _246335_at;

with _244901_at--_246334_at _246336_at--AFFX_r2_P1_cre_5_at;

ods exclude SimpleStats VarInformation;

run;

data correlations (drop = _TYPE_);

set correlations;

where abs(_246335_at) > 0.374 AND _TYPE_ = 'CORR'; /*0.374 was cutoff for P<0.05 in Proc Corr (noprint)*/

_NAME_ = substr(_NAME_, 2);

run;

proc sort data=correlations;

by _246335_at;

run;

/*Filter genes with significant correlations in original dataset*/

proc sql;

create table gds416 as

select *

from micro.gds416 as l left join

correlations as r

/*on (compress(l._,"_") = compress(r._NAME_,"_")); - other way of doing it*/

on (l._ = r._NAME_);

quit;

data gds416 (drop = _246335_at _NAME_); /*abbrev for only correlated variables or PAO*/

set gds416;

where _ = "246335_at" or _246335_at ne .;

run;

proc transpose data=gds416 out=gds416trans;

id _;

run;

data gds416transmod;

set gds416trans;

length Plant $20;

Plant1 = input(compress(_NAME_, '_'),best12.);

if 1348266 <= Plant1 < 1348268 then Plant = 'WT warm';

else if 1348268<= Plant1 < 1348270 then Plant = 'WT cold';

else if 1348270<= Plant1 < 1348272 then Plant = 'WT biological';

else if 1348272<= Plant1 < 1348274 then Plant = 'CRF2 overexp';

else if 1348274<= Plant1 < 1348276 then Plant = 'CRF3 overexp';

else if 1348276<= Plant1 < 1348278 then Plant = 'CZF1 overexp';

else if 1348278<= Plant1 < 1348280 then Plant = 'DEAR overexp';

else if 1348280<= Plant1 < 1348282 then Plant = 'ERF5 overexp';

else if 1348282<= Plant1 < 1348284 then Plant = 'HSFC overexp';

else if 1348284<= Plant1 < 1348286 then Plant = 'MYB4 overexp';

else if 1348286<= Plant1 < 1348288 then Plant = 'MYB7 overexp';

else if 1348288<= Plant1 < 1348290 then Plant = 'RAV1 overexp';

else if 1348290<= Plant1 < 1348292 then Plant = 'ZAT10 overexp';

else if 1348292<= Plant1 < 1348294 then Plant = 'ZF overexp';

drop Plant1;

run;

proc sort data=gds416transmod;

by Plant;

run;

/*MLR*/

title 'MLR';

proc reg data=gds416transmod plots=all;

model _246335_at = _244908_at--_246331_at _246343_at--_267646_at /selection=stepwise sle=0.05 sls=0.05;

ods select SelectionSummary;

run;

proc reg data=gds416transmod plots=all;

model _246335_at = _244908_at--_246331_at _246343_at--_267646_at /selection=forward sle=0.05;

ods select SelectionSummary;

run; /*could not do backward because too many genes, would never run correctly*/ /*forward and stepwise gave same results*/

/*I put these genes into a separate file to input*/

/*MLR gene identity*/

data mlrgenes;

infile 'resultsmlr.txt';

length probe $50;

input probe $;

probe = substr(probe,2);

run;

proc sql;

create table mlridentity as

select *

from affy2010 as affy inner join

mlrgenes as mlr

on (affy.probename = mlr.probe);

quit;

title 'MLR genes by function';

proc print data=mlridentity(drop=probe);

run;

/*interesting match*/

title 'Correlation of ATAF1 vs. PAO';

proc corr data=gds416transmod plots=matrix(histogram);

var _246335_at;

with _261564_at;

run;

title 'ANOVA of ATAF1 vs. PAO';

proc glm data=gds416transmod;

model _246335_at = _261564_at;

run;

title 'PAO and ATAF1 expression stats by Plant';

proc tabulate data=micro.gds416transmod;

class Plant;

var _246335_at _261564_at;

tables Plant,(_246335_at _261564_at)*(MEAN STD MIN MAX);

run;

ods pdf close;

**GSE5727 Code**

options nodate nonumber;

ods pdf file= 'gse5727microarray.pdf';

libname micro 'c:\labwork';

/*microarray import*/

proc import datafile='GSE5727abs.csv' out=micro.gds416 dbms=csv replace;

getnames=yes;

guessingrows=32767; /*to avoid data truncation*/

run;

proc transpose data=micro.gds416 out=micro.gds416trans;

id _;

run;

/*Affy probe names*/

data affy2010 (keep = probename genename description);

infile 'affy2010probenames.txt' dlm='09'x dsd firstobs=2;

length probename $40 x1 $100 x2 $100 x3 $100 genename $40 description $200;

input probename $ x1 $ x2 $ x3 $ genename $ description $;

run;

/*proc print data=affy2010;

where genename= 'AT3G44880'; PAO gene code

run;

var _246335_at probe name is our PAO*/

data micro.gds416transmod;

set micro.gds416trans;

length Plant $20;

Plant1 = input(compress(_NAME_, '_'),best12.);

Plant1 = (Plant1 - 1337000)/10;

if 29 <= Plant1 < 31 then Plant = 'WT pre-senescent';

else if 31<= Plant1 < 33 then Plant = 'WT senescent';

else if 33<= Plant1 < 35 then Plant = 'NahG';

else if 35<= Plant1 < 37 then Plant = 'ein2';

else if 37<= Plant1 < 39 then Plant = 'coi1';

drop Plant1;

run;

title 'Experimental Plant Frequencies';

proc freq data=micro.gds416transmod nlevels;

tables Plant;

run;

title 'PAO expression stats by Plant';

proc tabulate data=micro.gds416transmod;

class Plant;

var _246335_at;

tables Plant,_246335_at*(MEAN STD MIN MAX);

run;

/*PAO analysis by Plant*/

title 'Analysis of Variance (ANOVA) of PAO based on Plant';

proc glm data=micro.gds416transmod;

class Plant;

model _246335_at= Plant;

means Plant /tukey hovtest=BF;

lsmeans Plant;

output out=diagnostics p=ybar r=resid cookd=CookD;

run;

title2 'Diagnostics for ANOVA';

proc univariate data=diagnostics normal;

var resid;

qqplot /normal;

run;

proc sort data=micro.gds416transmod;

by Plant;

run;

/*Correlations*/

title 'Pearson Correlation for PAO vs. other genes';

proc corr data=micro.gds416transmod pearson nosimple noprint rank outp=correlations;

var _246335_at;

with _244901_at--_246334_at _246336_at--AFFX_r2_P1_cre_5_at;

ods exclude SimpleStats VarInformation;

run;

data correlations (drop = _TYPE_);

set correlations;

where abs(_246335_at) > 0.68 AND _TYPE_ = 'CORR'; /*0.68 was cutoff for P<0.05 in Proc Corr (noprint)*/

_NAME_ = substr(_NAME_, 2);

run;

proc sort data=correlations;

by _246335_at;

run;

/*Filter genes with significant correlations in original dataset*/

proc sql;

create table gds416 as

select *

from micro.gds416 as l left join

correlations as r

/*on (compress(l._,"_") = compress(r._NAME_,"_")); - other way of doing it*/

on (l._ = r._NAME_);

quit;

data gds416 (drop = _246335_at _NAME_); /*abbrev for only correlated variables or PAO*/

set gds416;

where _ = "246335_at" or _246335_at ne .;

run;

proc transpose data=gds416 out=gds416trans;

id _;

run;

data gds416transmod;

set gds416trans;

length Plant $20;

Plant1 = input(compress(_NAME_, '_'),best12.);

Plant1 = (Plant1 - 1337000) / 10;

if 29 <= Plant1 < 31 then Plant = 'WT pre-senescent';

else if 31<= Plant1 < 33 then Plant = 'WT senescent';

else if 33<= Plant1 < 35 then Plant = 'NahG';

else if 35<= Plant1 < 37 then Plant = 'ein2';

else if 37<= Plant1 < 39 then Plant = 'coi1';

drop Plant1;

run;

title 'data for /trans/';

proc print data=gds416trans (obs=1);

run;

title 'data for /transmod/';

proc print data=gds416transmod (obs=1);

run;

proc sort data=gds416transmod;

by Plant;

run;

proc contents data=gds416transmod;

run;

/*MLR*/

title 'MLR';

proc reg data=gds416transmod plots=all;

model _246335_at = _244901_at--_246320_at _246339_at--_267646_at /selection=stepwise sle=0.05 sls=0.05;

ods select SelectionSummary;

run;

proc reg data=gds416transmod plots=all;

model _246335_at = _244901_at--_246320_at _246339_at--_267646_at /selection=forward sle=0.05;

ods select SelectionSummary;

run; /*could not do backward because too many genes, would never run correctly*/ /*forward and stepwise gave same results*/

/*I put these genes into a separate file to input*/

/*MLR gene identity*/

data mlrgenes;

infile 'resultsmlr5727.txt';

length probe $50;

input probe $;

probe = substr(probe,2);

run;

proc sql;

create table mlridentity as

select *

from affy2010 as affy inner join

mlrgenes as mlr

on (affy.probename = mlr.probe);

quit;

title 'MLR genes by function';

proc print data=mlridentity(drop=probe);

run;

/*interesting match*/

title 'Correlation of ATAF1 vs. PAO';

proc corr data=gds416transmod plots=matrix(histogram);

var _246335_at;

with _261564_at;

run;

title 'ANOVA of ATAF1 vs. PAO';

proc glm data=gds416transmod;

model _246335_at = _261564_at;

run;

title 'PAO and ATAF1 expression stats by Plant';

proc tabulate data=micro.gds416transmod;

class Plant;

var _246335_at _261564_at;

tables Plant,(_246335_at _261564_at)*(MEAN STD MIN MAX);

run;

ods pdf close;

**GSE72050 Code**

options nodate nonumber;

ods pdf file= 'gse72050microarray.pdf';

libname micro 'c:\labwork';

/*microarray import*/

proc import datafile='GSE72050.csv' out=micro.gds416 dbms=csv replace;

getnames=yes;

guessingrows=32767; /*to avoid data truncation*/

run;

proc transpose data=micro.gds416 out=micro.gds416trans;

id _;

run;

/*Affy probe names*/

data affy2010 (keep = probename genename description);

infile 'affy2010probenames.txt' dlm='09'x dsd firstobs=2;

length probename $40 x1 $100 x2 $100 x3 $100 genename $40 description $200;

input probename $ x1 $ x2 $ x3 $ genename $ description $;

run;

/*proc print data=affy2010;

where genename= 'AT3G44880'; PAO gene code

run;

var _246335_at probe name is our PAO*/

data micro.gds416transmod;

set micro.gds416trans;

length Plant $20;

Plant1 = input(compress(_NAME_, '_'),best12.);

Plant1 = Plant1 - 1850500;

if 8 <= Plant1 < 10 then Plant = 'WT control';

else if 10<= Plant1 < 12 then Plant = 'WT drought';

else if 12<= Plant1 < 14 then Plant = 'VaNAC26-OE control';

else if 14<= Plant1 < 16 then Plant = 'VaNAC26-OE drought';

drop Plant1;

run;

title 'Experimental Plant Frequencies';

proc freq data=micro.gds416transmod nlevels;

tables Plant;

run;

title 'PAO expression stats by Plant';

proc tabulate data=micro.gds416transmod;

class Plant;

var _246335_at;

tables Plant,_246335_at*(MEAN STD MIN MAX);

run;

/*PAO analysis by Plant*/

title 'Analysis of Variance (ANOVA) of PAO based on Plant';

proc glm data=micro.gds416transmod;

class Plant;

model _246335_at= Plant;

means Plant /tukey hovtest=BF;

lsmeans Plant;

output out=diagnostics p=ybar r=resid cookd=CookD;

run;

title2 'Diagnostics for ANOVA';

proc univariate data=diagnostics normal;

var resid;

qqplot /normal;

run;

proc sort data=micro.gds416transmod;

by Plant;

run;

/*Correlations*/

title 'Pearson Correlation for PAO vs. other genes';

proc corr data=micro.gds416transmod pearson noprint nosimple rank outp=correlations;

var _246335_at;

with _244901_at--_246334_at _246336_at--AFFX_r2_P1_cre_5_at;

ods exclude SimpleStats VarInformation;

run;

data correlations (drop = _TYPE_);

set correlations;

where abs(_246335_at) > 0.70 AND _TYPE_ = 'CORR'; /*cutoff for P<0.05*/

_NAME_ = substr(_NAME_, 2);

run;

proc sort data=correlations;

by _246335_at;

run;

/*Filter genes with significant correlations in original dataset*/

proc sql;

create table gds416 as

select *

from micro.gds416 as l left join

correlations as r

/*on (compress(l._,"_") = compress(r._NAME_,"_")); - other way of doing it*/

on (l._ = r._NAME_);

quit;

data gds416 (drop = _246335_at _NAME_); /*abbrev for only correlated variables or PAO*/

set gds416;

where _ = "246335_at" or _246335_at ne .;

run;

proc transpose data=gds416 out=gds416trans;

id _;

run;

data gds416transmod;

set gds416trans;

length Plant $20;

Plant1 = input(compress(_NAME_, '_'),best12.);

Plant1 = Plant1 - 1850500;

if 8 <= Plant1 < 10 then Plant = 'WT control';

else if 10<= Plant1 < 12 then Plant = 'WT drought';

else if 12<= Plant1 < 14 then Plant = 'VaNAC26-OE control';

else if 14<= Plant1 < 16 then Plant = 'VaNAC26-OE drought';

drop Plant1;

run;

proc sort data=gds416transmod;

by Plant;

run;

proc contents data=gds416transmod;

run;

/*MLR*/

title 'MLR';

proc reg data=gds416transmod plots=all;

model _246335_at = _244901_at--_246329_at _246339_at--_267646_at /selection=stepwise sle=0.05 sls=0.05;

ods select SelectionSummary;

run;

proc reg data=gds416transmod plots=all;

model _246335_at = _244901_at--_246329_at _246339_at--_267646_at /selection=forward sle=0.05;

ods select SelectionSummary;

run; /*could not do backward because too many genes, would never run correctly*/ /*forward and stepwise gave same results*/

/*I put these genes into a separate file to input*/

/*MLR gene identity*/

data mlrgenes;

infile 'resultsmlr72050.txt';

length probe $50;

input probe $;

probe = substr(probe,2);

run;

proc sql;

create table mlridentity as

select *

from affy2010 as affy inner join

mlrgenes as mlr

on (affy.probename = mlr.probe);

quit;

title 'MLR genes by function';

proc print data=mlridentity(drop=probe);

run;

/*interesting match*/

title 'Correlation of ATAF1 vs. PAO';

proc corr data=gds416transmod plots=matrix(histogram);

var _246335_at;

with _261564_at;

run;

title 'ANOVA of ATAF1 vs. PAO';

proc glm data=gds416transmod;

model _246335_at = _261564_at;

run;

title 'PAO and ATAF1 expression stats by Plant';

proc tabulate data=micro.gds416transmod;

class Plant;

var _246335_at _261564_at;

tables Plant,(_246335_at _261564_at)*(MEAN STD MIN MAX);

run;

ods pdf close;
